# Cystinosis beyond kidneys: gastrointestinal system and muscle involvement

**DOI:** 10.1186/s12876-020-01385-x

**Published:** 2020-07-29

**Authors:** Rezan Topaloglu, Ayşe Gültekingil, Bora Gülhan, Fatih Ozaltin, Hülya Demir, Türkmen Çiftci, Numan Demir, Çağrı Mesut Temucin, Aysel Yuce, Okhan Akhan

**Affiliations:** 1 Division of Pediatric Nephrology, Hacettepe University School of Medicine, 06100 Sıhhiye, Ankara, Turkey; 2 Department of Pediatrics, Hacettepe University School of Medicine, Ankara, Turkey; 3 Nephrogenetics Laboratory, Hacettepe University School of Medicine, Ankara, Turkey; 4 Division of Pediatric Gastroenterology, Hacettepe University School of Medicine, Ankara, Turkey; 5 Department of Radiology, Hacettepe University School of Medicine, Ankara, Turkey; 6 Hacettepe University School of Physiotherapy, Ankara, Turkey; 7 Department of Neurology, Hacettepe University School of Medicine, Ankara, Turkey

**Keywords:** Cystinosis, Extrarenal, Gastrointestinal, Complication

## Abstract

**Background:**

Cystinosis is a multisystemic disease resulting from cystine accumulation primarily in kidney and many other tissues. We intended to study the evolution of less commonly seen extrarenal complications of cystinosis in a group of patients who have periods without cysteamine treatment.

**Methods:**

Gastrointestinal and muscular complications of cystinosis were studied in a group of 21 patients.

**Results:**

Twenty one patients were included in the study. Among them, 14 were homozygous and 3 were compound heterozygous for *CTNS* mutations. The median age of diagnosis was 15 months (range; 5 months-14 years) and the mean age at last visit was 11.3 ± 6.5 years. Nine patients (42%) had end stage renal disease at a mean age of 10.6 years (6.5–17 years). Abdominal ultrasonography and portal vein doppler ultrasonography were performed in19 patients, 14 of them (74%) had hepatomegaly, 10 patients (53%) had granular pattern or heterogeneity of liver. Only one patient had high transaminase levels and liver biopsy showed cystine crystals in the liver. Eleven patients (58%) had borderline or increased portal vein minimum and maximum flow velocities. One patient had CK level of 9024 U/L and electromyographic study showed active myopathic involvement. Two patients were found to have gastroesaphageal reflux only and 4 patients were found to have esophageal remnants in addition to reflux.

**Conclusions:**

In addition to renal functions, extrarenal organs may be affected from cystine accumulation even in childhood, especially in patients who are incompliant to treatment, resulting in complications such as swallowing difficulty, hepatomegaly and portal hypertension.

## Background

Cystinosis is a rare autosomal recessive lysosomal storage disorder which is characterized by deficient excretion of cystine from lysosome to cytoplasm resulting in cystine crystal accumulation in various organs including kidneys, cornea, bone marrow, thyroid gland, liver, spleen and muscles [1].

It is caused by bi-allelic mutations in the *CTNS* gene encoding the lysosomal cystine transporter, cystinosin [2–4]. Wide spectrum of mutations of *CTNS* gene have been shown in different populations and in Turkish patients [5].

Cystine accumulation takes place in renal tubuli first, leading to renal Fanconi syndrome which is responsible for all of the early manifestations of the disease like polyuria, polydipsia, rickets, feeding difficulties and growth retardation [1, 6]. Eventually, cystine also accumulates in glomeruli leading glomerular proteinuria and glomerulosclerosis. Later, glomerular filtration rate declines with time, serum creatinine levels start to rise after 5 years of diagnosis and end stage renal disease (ESRD) necessitating renal replacement therapy is seen around 7–12 years old unless treatment is initiated [1, 6, 7].

Successful treatment of cystinosis depends on early diagnosis and effective treatment and consists of supportive therapy and specific therapy with cysteamine [1, 6]. Cysteamine decreases intracellular cystine content by 90% and delays deterioration of renal function and promotes growth [8–11].

Even if renal deterioration can be taken under control with cysteamine, extrarenal accumulation of cystine continues and different aspects of cystinosis can be seen as more patients reach to adult ages [1, 6]. Although opthalmic and endocrine system complications are seen frequently, many other systems are affected also like gastrointestinal and muscular systems [1, 4, 6, 12]. In terms of gastrointestinal system invovement, hepatomegaly is a common finding usually without liver dysfunction [13, 14]. Cystine crystal accumulation activates Kupffer cells and secreted vasoactive substances cause vasoconstriction in microcirculation resulting in portal hypertension [13–15]. Other gastrointestinal complications are feeding difficulty and anorexia. Direct accumulation of cystine in lamina propria and muscles can also result in oromotor dysfunction and dysphagia [16–18]. Muscular cystine accumulation is also found in skeletal muscles especially in thenar and hypothenar muscles of the hand and respiratory muscles causing restrictive type of respiratory insufficiency [2, 19–21]. In this study, we aimed to investigate gastrointestinal and muscular system complications of cystinosis.

## Methods

### Design and setting

Twenty one historical cystinosis patients who were followed up at Pediatric Nephrology Department of Hacettepe University were included in this prospective study. Cystinosis was diagnosed on the basis of observation of corneal cystine crystals via eye examination and/or an elevated leukocyte cystine level (> 2 nmol half-cystine per mg protein) and/or *CTNS* gene mutation(s). Adherence to cysteamine treatment was determined by the physician according to the answer given to the following question: how do you assess your adherence to cysteamine: good, quite good, or period(s) without treatment. All of the patients in the study had period(s) without treatment. Medical records were evaluated and age at diagnosis, consanguinity between parents, clinical signs and symptoms at the time of diagnosis, initiation of cysteamine treatment, duration of clinical follow up, serum creatinine and glomerular filtration rates were recorded (Table 1).

**Table 1:** General clinical characteristics of the patients^a^

Pt No	Age (years)	Sex	Age at Dx (months)	RF Stage /RRT	GOR	HM	SM	Granularity of liver	Increased portal flow	Endoscopy	CK (U/L) (Ref; 0–100)	LCL	***CTNS*** mutation
1	9	F	11	2	–	–	–	–	–	N/A	85	1.39	c.829 dup (p.Thr277Asnfs^a^19)/ 518A > G (pY173C)
2	9	M	12	4	–	+	–	+	–	N/A	53	2.06	N/A
3	15	F	14	5,Tx	–	+	+	+	+	N/A	42	3.06	c.681G > A (p.Glu227Glu)/ c.1015G > A (p.Gly339Arg)
4	11	M	10	5,PD	–	+	+	+	+	N/A	9024	1.05	c.18_21 del (p.Thr7PhefsTer7) (H)
5	13	M	15	GFR > 90	–	–	–	–	–	N/A	71	2.67	c.451A > G (p.Arg151Gly)/ c.1015G > A (p.Gly339Arg)
6^b^	16	M	24	5,Tx	–	–	–	–	–	N/A	87	1.5	c.1015G > A (p.Gly339Arg) (H)
7^b^	6	F	16	2	–	–	–	–	–	N/A	78	4.1	c.1015G > A (p.Gly339Arg) (H)
8	16	F	12	2	–	+	+	+	+	Salt and pepper appearance in antrum	42	.64	c.518A > G (pY173C) (H)
9	8	M	54	3	–	+	–	–	+	N/A	119	1.67	c.140 + 1G > T (H)
10	9	F	60	5,Tx	–	–	–	+	+	N/A	60	3.62	c.664C > T (p.Gln222X) (H)
11	16	M	12	5,HD	+	+	+	–	+	N/A	46	4.90	c.62-1083_c.551del10217 (H)
12	21	M	12	5,PD	+	+	+	–	+	Portal gastropathy, bile reflux	97	1.5	c.140 + 1G > T (H)
13	15	M	18	5,Tx	N/A	+	+	+	–	N/A	55	4.20	c.1015G > A (p.Gly339Arg) (H)
14	15	M	168	2	+	+	+	+	+	N/A	61	8.30	c.451A > G (p.Arg151Gly) (H)
15	4	M	48	2	+	+	+	–	–	N/A	73	4.2	c.18_21 del (p.Thr7PhefsTer7) (H)
16^c^	5	F	18	GFR > 90	–	+	–	–	–	N/A	59	2.63	N/A
17^c^	1	M	5	GFR > 90	N/A	N/A	N/A	N/A	N/A	N/A	127	1.85	N/A
18	29	F	106	5,Tx	+	+	–	+	+	N/A	37	1.12	N/A
19	4	M	54	3	+	+	+	+	+	Normal	86	15.75	c.681G > A (p.Glu227Glu) (H)
20	10	M	14	5,PD	–	+	+	+	+	Edema in corpus, antrum	217	2.82	c.141-22A > G (H)
21	6	M	12	2	–	N/A	N/A	N/A	N/A	N/A	46	3.8	c.681G > A (p.Glu227Glu) (H)

^a^All of the patients had period(s) without cysteamine treatment

^b^Pt. number 6 and 7 are siblings

^c^Pt. number 16 and 17 are siblings

*Abbreviations*: *CK* creatine kinase, *Dx* diagnosis, *F* female; *GOR* gastroesophageal reflux, *H* homozygous, *HD* hemodialysis, *HM* hepatomegaly, *M* male, *N/A* not available, *LCL* Leukocyte cystine level (nmol half-cystine/mg.protein) (reference range: < 0.2), *PD* peritoneal dialysis, *Pt* patient, *RF* renal failure, *RFT* respiratory function test, *RRT* renal replacement therapy, *SM* splenomegaly, *Tx* transplantation

At last visit, creatine kinase (CK) were measured in all 21 patients and nerve conduction studies (NCS) and, needle electromyographic (EMG) examination were performed in 8 patients who are older than 10 years. Pharyngoeosaphagography with barium contrast was performed in twenty patients and swallowing function was evaluated in solid, semi-solid and liquid phases, especially chewing movements, elevation of palate, transition to pharyngeal phase, state of arytenoids and vocal cords during swallowing, gastroesophageal reflux and presence of remnants were noted. Videofloroscopy test procedure; Standard modified barium test was applied to patients. The patients were evaluated as thin liquid, pudding consistency and solid consistency. In the test procedure, 3 ml liquid barium for liquid consistencies, 3 ml pudding barium for pudding consistency, and standard test biscuit dipped in barium for solid consistency were used. During the test, oral phase, pharyngeal phase, esophagal phase were evaluated in detail. For the oral phase; oral transit time, presence of residue was noted. In the pharyngeal stage, delay in swallowing reflex, pharyngeal transition time, penetration, aspiration and nasal regurgitation were evaluated. The presence of aspiration and penteration was assesed using the penetration aspiration scale. In the esophagal phase, esophageal transition time, backflow, reflux and dysmotility were evaluated.

At last visit, serum aminotransferase levels were measured and hepatobiliary and splenic ultrasonographic and portal doppler ultrasonographic studies were performed, direction of portal blood flow, presence of collateral veins, portal vein diameter, portal vein minimum and maximum velocities were measured. Upper gastrointestinal endoscopic study was performed in 5 patients to evaluate portal hypertension in patients with splenomegaly.

Cysteamine treatment was started to all patients at diagnosis, average inital cysteamine dose was 60 mg/kg/day. The patients were grouped into two as patients who had leukocyte cystine level (LCL) at or above 2 nmol half cystine/mg protein and below 2 nmol half cystine/mg protein.

### Genetic analyses

All genetic testing was performed at the Hacettepe University Nephrogenetics Laboratory. Briefly, DNA was extracted from peripheral blood following the standard phenol-chloroform protocol. All exons of the *CTNS* gene together with their adjacent intron junctions were analyzed via direct sequencing using BigDye v.3.1 chemistry and an ABI3130 genetic analyzer (Applied Biosystems, Foster City, CA). The National Center for Biotechnology Information transcript variant 1 of *CTNS* (NM_001031681.2), corresponding to ENSEMBL transcript ENST00000381870.7, was used as a reference sequence. Analysis of the *CTNS* gene was not performed in patients whose parents did not consent.

### Statistical analyses

Descriptive statistical analysis methods were used to evaluate demographic and clinical data. Mean, median, SD, and interquartile range (IQR) were calculated for numeric variables. Frequency tables were used to describe categorical data. The Mann–Whitney U test or independent samples t test was used to compare two independent samples. All data were analyzed using IBM SPSS Statistics for Windows v.21 (IBM Corp., Armonk, NY).

The study protocol was approved by the Hacettepe University Ethics Committee (HEK 09/134–42) and carried out in accordance with the Declaration of Helsinki. Written informed consent was obtained from the patients and/or parents of all patients.

## Results

### Patient characteristics

Twenty one patients (14 boys and 7 girls) were included in the study. Among them, 17 patients consented genetic testing, 14 were homozygous and 3 were compound heterozygous for *CTNS* mutations. To calculate allele frequencies only one patient from each family was considered (even if a family had multiple affected individuals) (i.e. 16 patients, 32 alleles). The most common mutation was c.1015G > A [p.Gly339Arg] which was present in 6 alleles (19%) followed by c.681G > A [p.Glu227Glu] (5 alleles; 15.6%). The median age of cystinosis diagnosis was 15 months (range; 5 months-14 years) and the mean age at last visit was 11.3 ± 6.5 years. In total, 14 patients (66.7%) were diagnosed before 2 years of age and 8 patients were diagnosed at or before 1 year of age. The mean period of follow up after diagnosis of cystinosis was 9.5 years (range; 0.5–20 years). Parental consanguinity was observed in 10 families (48%). Two patients (14,2%) had cystinotic siblings in the family. The most common symptom at diagnosis was polyuria (14 patients; 66.7%). Other symptoms at diagnosis were as follows; growth retardation (13 patients, 62%), polyuria (10 patients, 48%), vomiting (7 patients, 33%), inability to walk (3 patients, 14%). At the time of diagnosis, the most common laboratory abnormalities were metabolic acidosis (17 patients, 81%) and proteinuria (17 patients, 81%). Median LCL of the whole group was 2.7 (IQR; 1.5–4.1) nmol half cystine/mg protein.

#### Renal functions

At the time of the study, three patients (14.3%) had glomerular filtration rate (GFR) above 90 mL/min/1.73m^2^ and nine patients (42%) had ESRD at a mean age of 10.6 years (6.5–17 years) and needed renal replacement therapy (hemodialysis, peritoneal dialysis or renal transplantation) (Table 1). Five of nine patients had renal transplantation at a mean age of 15.3 years (range; 9–29 years), all but one have normal graft function. One patient experienced rejection due to inadherence to immunosuppressive drugs. Other patients were in various stages of chronic kidney disease.

#### Gastrointestinal system findings

Abdominal ultrasonography and portal vein doppler ultrasonography were performed in19 patients (two patients did not give informed consent), 14 of them (74%) had hepatomegaly, 10 patients (53%) had granular pattern or heterogeneity of liver. In cohort, only one patient had high transaminase levels and liver biopsy showed cystine crystals in the liver. The most common alleles in patients with hepatomegaly and/or granular pattern or heterogeneity of liver are c.18_21 del (p.Thr7PhefsTer7) and c140 + 1G > T (4 alleles each, 16.7%).

Eleven patients (58%) had borderline or increased portal vein minimum and maximum flow velocities (minimum 19–35 cm/sec, maximum 35–51 cm/sec), all patients had hepatopedal flow in portal vein and none of them had collateral veins. Portal vein diameters ranged between 7,5–12 mm, 10 patients (53%) had increased portal vein diameter according to age. Ten patients (53%) were found to have splenomegaly, 5 of these patients (26%) also had increased portal vein diameter. Endoscopic evaluation was performed in these 4 patients with splenomegaly but no sign of portal hypertension was observed. The most common allele in patients with splenomegaly is c.18_21 del (p.Thr7PhefsTer7) (4 alleles, 20%).

**Fig. 1:**
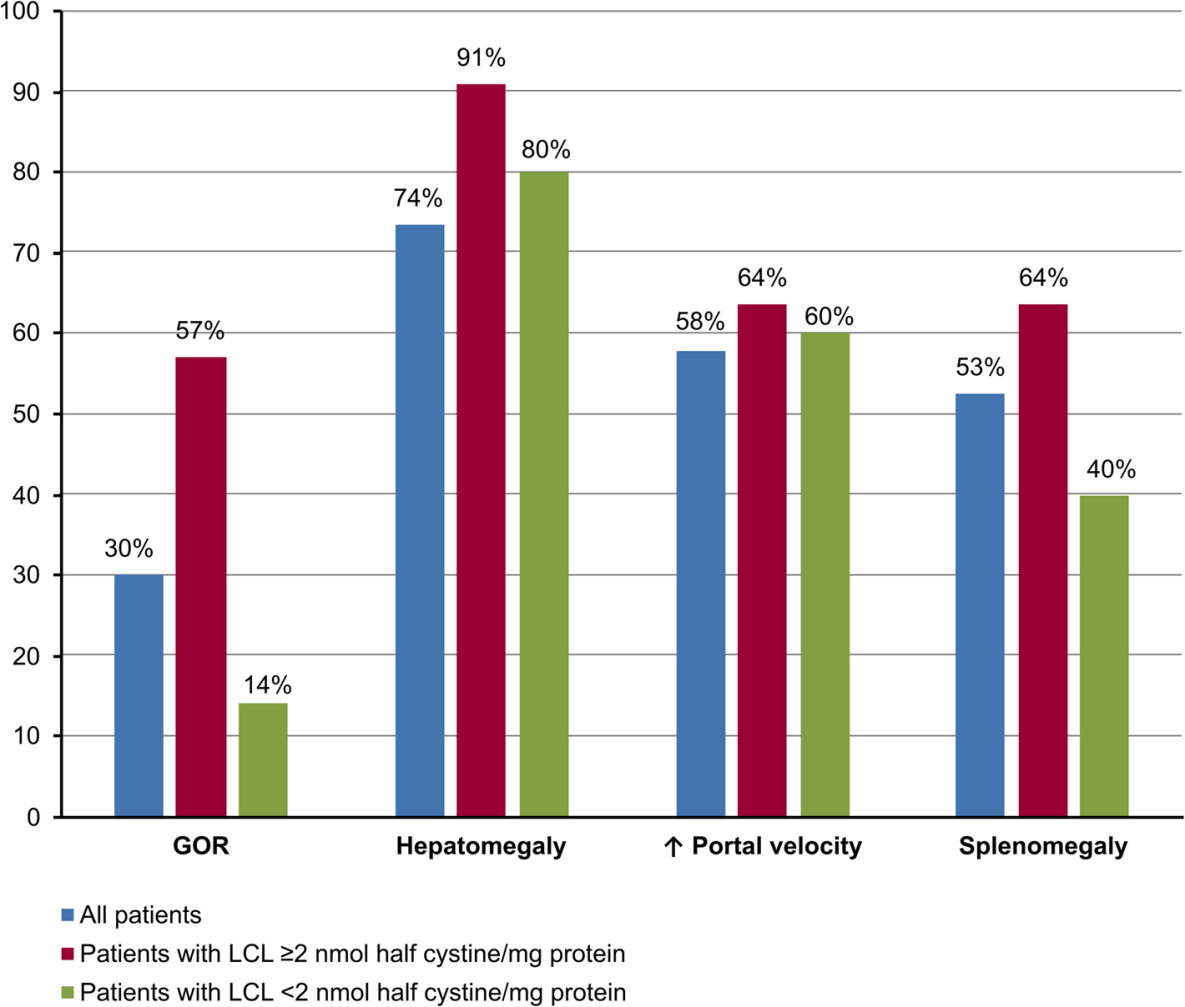
Percentage of the patients with gastrointestinal complications according to leucocyte cystine level

The patients who had LCL at or above 2 nmol/mg had gastrointestinal complications more frequently than the patients with LCL below 2 nmol/mg, but this difference was not statistically significant (*p* = 0.625) (Fig. 1).

#### Muscular and swallowing functions

In our group, patient no.4 had minimal muscle weakness on physical examination which had been attributed to another sign of cystinosis. However, he had CK level of 9024 U/L (patient no. 4) (Table 1). NCS that performed at median, tibial and sural nerves were normal. However, needle EMG examination showed active myopathic involvement at both proximal (biceps brachii and iliopsoas) and distal (tibialis anterior) muscles. There was short duration, low amplitude, polyphasic motor unit potentials with early recruitment on voluntary activity and abnormal spontaneous activities (fibrillation and positive sharp waves, complex repetitive discharges) which were more prominent at proximal muscles. He was inadherent to the cysteamine treatment. The genetic analysis of this patient showed homozygous c.18_21 del [p.Thr7Phefs*7] mutation. Other patients had normal CK levels and normal electromyography (EMG) (eight patients older than 10 years).

Modify barium swallow test; Except for the 5-month-old baby, all patients received a modified barium swallowing test procedure. None of the patients had any problem in the oral and pharyngeal phase of swallowing in all test consistencies. Aspiration and penetration were not observed. No residue was observed in the oral and pharyngeal regions. In esophageal phase examination of swallowing, visible reflux was detected in 2 patients (10%). In 4 patients (20%), esophageal motility problem and delay in esophageal transition period were observed. The mean age of these patients was 15 years (4–29 years), 3 of them had ESRD (Table 1). Fifty seven percent of the patients who had LCL at or above 2 nmol/mg had swallowing dysfunction however only 14% of the patients who had less than 2 nmol/mg of LCL had swallowing dysfunction (*p* = 0.545).

## Discussion

Cystinosis affects renal tubuli and causes renal failure in the first decade if left untreated. However renal transplantation and cysteamine treatment prolonged the survival and changed the appearance of disease from a renal disorder to a multisystemic metabolic disease. Therefore, analysis of other systemic complications of cystinosis is also crucial. In this study, we have investigated extrarenal complications and impact of treatment on progression of these complications.

Age of the patients in the study ranged between 6 months to 29 years allowing to see complications in different age groups. Almost half of the parents of patients were related to each other and consanguinity between parents was more frequent than average of Turkish population (21%) [22]. More than half of the patients were diagnosed before 2 years of age and 8 patients were diagnosed at or before 1 year of age which could be considered fairly early [1].

Patients were followed up approximately 10 years on average, GFR and creatinine levels were normal in early childhood as expected. Although half of the patients were diagnosed before 2 years of age and cysteamine treatment was started to all the patients at time of diagnosis, about half of patients had ESRD at 10.6 years. This may be due to the inadherence to treatment and high LCL levels. This finding is consistent with literature as it was shown that cysteamine therapy slows the renal progression of the disease especially if it was started before age 5 and age of renal failure directly correlates with compliance to therapy which is measured with LCL [23, 24].

Among 5 transplanted patients, only one patient had rejection after transplantation and that was due to inadherence to immunosuppressive regimen, other patients who had transplantation did well afterwards. None had rejection or recurrence concordant with previous studies [1, 5, 25, 26].

The most frequent (50–70%) of *CTNS* mutations in Northen Europe is a large 57-kb which constitutes 75% of the mutated alleles in patients of Northern European descent with cystinosis [27]; however, the spectrum of the mutations varies according to geography. Traveling from Northern Europe and America to the Southern Hemisphere, the frequency of the 57-kb deletion decreases. Mason et al. [28] studied *CTNS* mutations in Italian patients with cystinosis and observed that the 57-kb deletion was present in only 17% of the patients. Topaloglu et al. studied the genetic spectrum in a large group of pediatric patients from a national cystinosis registry and confirmed that none of the patients had thee 57-kb *CTNS* deletion. In that study, the most common alleles in Turkey is c.681G.A (p.E227E; 31%), c.1015G.A (p.Gly339Arg; 22%) and c.18_21 del (p.Thr7Phefs*7; 14%) [29]. Consistent with this result, in our study, the most common mutation was c.1015G > A [p.Gly339Arg] (20%) followed by c.681G > A [p.Glu227Glu] (16.7%).

Myopathic complications of cystinosis are expected to be seen in adulthood [30]. Recently, Brodin-Sartorius reported that 22 out of 86 adult cystinosis patients had myopathy [23]. In our study, an 11 year old patient was found to have high CK levels and active myopathic findings on electromyographic examination, which is fairly early. He is a carrier of c.18_21 del [p.Thr7Phefs*7] on *CTNS* gene which is also a common mutation in Turkey. This deletion was first defined by Town et al. and causes a frameshift at exon 7 and forms a stop codon. He did not have any complaints of weakness however myopathy could be found before patient had symptoms as showed by Vester et al. [19]. Besides to the inadherence to the cysteamine treatment, this involvement may be associated with the severity of the mutation. This case also highlighted that muscular involvement can be also observed in pediatric patients in earlier ages according to the severity of the mutation and adherence to the cysteamine treatment. Swallowing dysfunction is also said to be seen in adolescent ages especially increasing after 16 years [18, 19]. In different adults series, swallowing impairment was observed in 24–73% of the patients [18, 23]. In our pediatric group, approximately 30% of the patients had swallowing impairment. The mean age of patients was 15 years. It is important to diagnose this complication in younger patients because adequate nutrition and growth is very essential in this group of patients. Beyond adequate nutrition, swallowing is also important in the prognosis of the cystinosis patient. Brodin-Sartorius investigate the prognosis of cystinosis in a late adolescents and adults. They separated the patients into three groups: patients who started cysteamine therapy before 5 years of age, after 5 years of age and those who were not treated before development of ESRD. In the group treated after 5 years of age, the most common cause of death were respiratory distress due to swallowing impairment and neurological reasons. Our study showed that swallowing impairment can be observed in younger ages in patients who were inadherent to therapy [23].

The data regarding to the gastrointestinal system involvement pediatric cystinosis patients is scarce in the literature. Hepatomegaly without liver failure is a common finding in cystinosis, it is a result of cytokine reaction to cystine crystals and collagen deposition without accompanying fibrosis and bridging necrosis [1, 5, 13–15], which is also common in our group, hepatomegaly was found in three quarters of patients and change in liver pattern in more than half of the patients. Cystine accumulation in liver also causes secretion of vasoactive substances that results in vasoconstriction and portal hypertension [1, 5, 13, 14]. More than half of our patients had splenomegaly and also had increased portal vein diameter leading to possible diagnosis of portal hypertension however no sign of portal hypertension was found in endoscopic studies. Nevertheless these patients should be followed up as splenomegaly together with increased portal vein diameter may progress to portal hypertension with development of reverse flow and collaterals incresing the morbidity and mortality of the patients [31].

Gastrointestinal complications were seen less in patients who had appropriate treatment with low levels of LCL. However this did not reach to statistical significance. This may be due to relative small number of the patients. This support the fact that cysteamine treatment decrease cystine accumulation in many organs other than kidneys and cysteamine treatment slows down the progression of disease not only in kidneys but also extrarenal organs as well [23, 31].

## Conclusion

We concluded that in addition to renal functions, many extrarenal organs may be affected from cystine accumulation even in childhood especially in patients who are incompliant to therapy, resulting in complications such as swallowing difficulty, respiratory problems, hepatomegaly and portal hypertension. Although our results are not representative for well-treated patiens, children with cystinosis should be followed up in a multidisciplinary approach besides focusing on renal complications.

## Data Availability

The datasets used and/or analysed during the current study are available from the corresponding author on reasonable request.

## Abbrevıatıons

CK: Creatine kinase
Dx: Diagnosis
EMG: Electromyography
ESRD: End stage renal disease
F: Female
GFR: Glomerular filtration rate
GOR: Gastroesophageal reflux
H: Homozygous
HD: Hemodialysis
HM: Hepatomegaly
IQR: Interquartile range
LCL: Leukocyte cystine level
M: Male
N/A: Not available
PD: Peritoneal dialysis
Pt: Patient
RF: Renal failure
RFT: Respiratory function test
RRT: Renal replacement therapy
SM: Splenomegaly
Tx: Transplantation
