# Correction to: Cystinosis beyond kidneys: gastrointestinal system and muscle involvement

**DOI:** 10.1186/s12876-022-02166-4

**Published:** 2022-04-05

**Authors:** Rezan Topaloglu, Ayşe Gültekingil, Bora Gülhan, Fatih Ozaltin, Hülya Demir, Türkmen Çiftci, Numan Demir, Çağrı Mesut Temucin, Aysel Yuce, Okhan Akhan

**Affiliations:** 1 Division of Pediatric Nephrology, Hacettepe University School of Medicine, 06100 Sıhhiye, Ankara, Turkey; 2 Department of Pediatrics, Hacettepe University School of Medicine, Ankara, Turkey; 3 Nephrogenetics Laboratory, Hacettepe University School of Medicine, Ankara, Turkey; 4 Division of Pediatric Gastroenterology, Hacettepe University School of Medicine, Ankara, Turkey; 5 Department of Radiology, Hacettepe University School of Medicine, Ankara, Turkey; 6 Hacettepe University School of Physiotherapy, Ankara, Turkey; 7 Department of Neurology, Hacettepe University School of Medicine, Ankara, Turkey

## Correction to: BMC Gastroenterology (2020) 20:242 10.1186/s12876-020-01385-x

After publication of this article [1], the authors reported that the author name Ayşe Gültekingil was incorrectly written as Ayşe Gültekingil Keser. The original article [1] has been updated.
